# Epigenetic Changes and Chromatin Reorganization in Brain Function: Lessons from Fear Memory Ensemble and Alzheimer’s Disease

**DOI:** 10.3390/ijms232012081

**Published:** 2022-10-11

**Authors:** Brigitte van Zundert, Martin Montecino

**Affiliations:** 1 Institute of Biomedical Sciences, Faculty of Medicine and Faculty of Life Sciences, Universidad Andres Bello, Santiago 8370186, Chile; 2 CARE Biomedical Research Center, Santiago 8330005, Chile; 3 Millennium Institute Center for Genome Regulation CRG, Santiago 8370186, Chile

**Keywords:** epigenetic regulation in brain, chromatin organization during cognition, mechanisms of synaptic plasticity, epigenetic editing in neurodegenerative diseases

## Abstract

Healthy brain functioning in mammals requires a continuous fine-tuning of gene expression. Accumulating evidence over the last three decades demonstrates that epigenetic mechanisms and dynamic changes in chromatin organization are critical components during the control of gene transcription in neural cells. Recent genome-wide analyses show that the regulation of brain genes requires the contribution of both promoter and long-distance enhancer elements, which must functionally interact with upregulated gene expression in response to physiological cues. Hence, a deep comprehension of the mechanisms mediating these enhancer–promoter interactions (EPIs) is critical if we are to understand the processes associated with learning, memory and recall. Moreover, the onset and progression of several neurodegenerative diseases and neurological alterations are found to be strongly associated with changes in the components that support and/or modulate the dynamics of these EPIs. Here, we overview relevant discoveries in the field supporting the role of the chromatin organization and of specific epigenetic mechanisms during the control of gene transcription in neural cells from healthy mice subjected to the fear conditioning paradigm, a relevant model to study memory ensemble. Additionally, special consideration is dedicated to revising recent results generated by investigators working with animal models and human postmortem brain tissue to address how changes in the epigenome and chromatin architecture contribute to transcriptional dysregulation in Alzheimer’s disease, a widely studied neurodegenerative disease. We also discuss recent developments of potential new therapeutic strategies involving epigenetic editing and small chromatin-modifying molecules (or epidrugs).

## 1. Introduction

Brain function in mammals requires tight control of gene expression in all neural cells. Moreover, processes mediating learning and memory involve the establishment of cell engrams that support efficient neuron connectivity, which is strongly based on the ability of these cells to express proper gene profiles. Accumulating evidence during the last four decades demonstrates that epigenetic mechanisms modulating dynamic changes in chromatin organization are critical components during the regulation of gene transcription in response to physiological cues (for reviews see [1,2]). Moreover, aberrant epigenetic mechanisms are associated with pathological brain processes, including neurodegenerative disorders such as Alzheimer’s disease (AD) (for reviews see [1,3]). Recent genome-wide studies using postmortem non-diseased human brains also support the intriguing possibility that AD risk variants located in enhancer regions may change gene expression by altering the interaction between promoters and enhancers [4,5]. Together, these findings support the raising concept that the onset and/or progression of several brain-related pathologies may be directly associated with a reduced ability of neural cells to establish and sustain a chromatin configuration that efficiently permits the required gene expression profile. More importantly, the data point to a number of genes and regulatory pathways in brain cells that may be used as potential new therapeutic targets and diagnostic tools. 

Here, we overview recent relevant results in the field supporting the role of the chromatin organization and specific epigenetic mechanisms during the control of gene transcription in neural cells. Special consideration is dedicated to reviewing data generated by investigators using the fear-conditioning paradigm to study learning and memory, as well as animal models specifically developed to address basic molecular mechanisms in Alzheimer’s disease. 

## 2. Mechanisms of Epigenetic Control in Mammals

In eukaryotic cells, the genome is organized through a complex structure of proteins and DNA named chromatin. This organization is a key component during the regulation of gene expression as the cells can modify the level of compaction of chromatin and hence alter the access of the transcriptional machinery to specific genomic sequences. The fundamental unit of chromatin is the nucleosome, which includes a DNA segment of approximately 147 bp in length that is wrapped around an octamer of histone proteins (two each of histones H2A, H2B, H3 and H4) [6,7]. The N-terminal sequence of these histone proteins (histone tales) protrudes outside the limits of the nucleosome particle and provides a surface for recognition by, and for subsequent interaction with, proteins that regulate transcription [8,9,10]. Moreover, residues (e.g., lysine residues) within these histone tails can be targets for enzyme-mediated post-translational modifications, hence, altering the chemical environment of particular regions of the chromatin fiber and potentially affecting gene expression in cells [9]. Importantly, genomic DNA can be also enzymatically modified, affecting also the degree of compaction of chromatin, and hence, contributing to regulating transcription [11,12]. 

**Figure 1:**
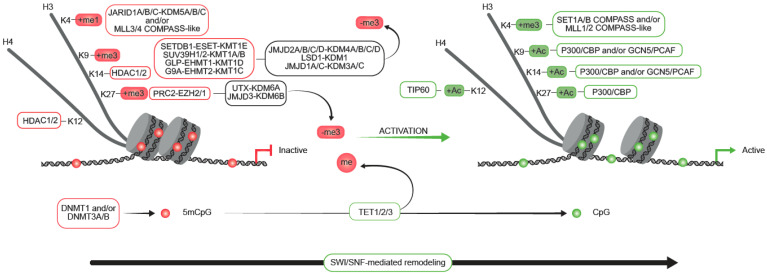
Epigenetic changes during transcriptional activation in mammalian cells. The diagram depicts epigenetic marks associated with transcriptional activation or repression of a gene promoter. Repression-associated epigenetic marks are shown in red (poorly acetylated H4K12 and H3K14, H3K4me1, H3K9me3, H3K27me3 and 5mCpG (for methylated DNA)) and active transcription marks are shown in green (H4K12Ac, H3K4me3, H3K9Ac, H3K14Ac, H3K27Ac, and CpG (for unmethylated DNA)). Enzymes that can mediate deposition (“writers”) and elimination (“erasers”) of these marks are also indicated. The transcriptional start site (TSS) of the gene in active (green) and repressed (red) conditions is also shown. Gray cylinders represent nucleosomes positioned over the promoter region of the active (relaxed disposition) and repressed (compacted disposition) gene.

Nuclear proteins that regulate chromatin organization and transcription are critical players during cell responsiveness to external physiological cues. Among them, we find the large group of enzymes that can catalyze a wide number of “histone post-translational modifications” (HPTMs), a set of marks that together constitute a principal “epigenetic” mechanism [9,10,13]. HPTMs can function as docking sites on the chromatin surface that can be recognized by specific nuclear proteins (“epigenetic readers”) that contain complementary high-affinity domains (e.g., chromo-domains interact with methylated histone lysine residues or bromo-domains that recognize acetylated histone lysine residues) [10,14]. In addition, a significant number of nuclear protein complexes that are capable of mediating deposition (“epigenetic writers”) or elimination (“epigenetic erasers”) of HPTMs in eukaryotic cells (see Figure 1) include core subunits that are evolutionarily conserved [15,16,17]. Together, these findings provide strong support to the idea that the contribution of these nuclear complexes to gene expression control is conserved across the species.

Acetylation of histone lysine residues was one of the first reported HPTMs in eukaryotic cells [18]. Enzyme-mediated transferring of an acetyl group from the cellular substrate acetyl CoA to a histone protein neutralizes the positive charge of the acceptor lysine residue and can decrease the affinity between acetylated histones and the negatively charged DNA within the nucleosome particle. This in turn may result in de-compaction of chromatin fibers that favors transcriptional activity [9]. The enzymes that mediate this acetylation reaction, that were initially discovered through genetic-based screen analyses, are known as Histone Acetyltransferases (HATs) and have been shown to be critical regulatory components of nuclear complexes that promote transcription [19,20]. Among them we can find the transcription coactivators P300 (E1A Associated protein P300) and CBP (CREB (Cyclic AMP Response Element Binding Protein binding) Binding Protein), GCN5 (General Control Non-Depressible 5), PCAF (P300/CBP Associated Factor) and TIP60 (TAT Interacting Protein 60), that are recruited to target promoters and enhancer sequences by transcription factors to produce high-chromatin acetylation [20,21]. Histone acetylation is a highly dynamic mark on the chromatin as it can be rapidly removed by the activity of a large family of specialized proteins containing histone deacetylase activity (HDACs) [19]. These HDACs are also recruited to regulatory regions of the genome (Figure 1) by sequence-specific transcription factors that, in this case, function as transcriptional repressors.

Genetic studies in model systems also permitted the identification of nuclear protein complexes that control gene transcription by modifying the profile of methylated lysine residues in histones associated with chromatin. Among them are the Polycomb Group (PcG) and the Trithorax Group (TrxG) complexes, which can mediate inhibition and activation of transcription, respectively [22]. One signature property of the evolutionarily conserved PcG complexes PRC1 and PRC2 (Polycomb Repressive Complex 1 and 2, respectively) is to mediate the formation of repressed chromatin. In mammals, PRC2 is included as a principal subunit of the highly conserved protein Enhancer of Zeste Homolog 2 or 1 (EZH2/EZH1) [16,23]. EZH2 is the main catalytic component of PRC2 and mediates tri-methylation of the lysine 27 residue of histone H3 (H3K27me3), a modification that is associated with transcriptionally silent chromatin (Figure 1) [10,16].

Mammalian TrxG complexes, including COMPASS (Complex of Proteins Associated with SET1A/B) and the Mixed Lineage Leukemia (MLL1 to 5)-containing COMPASS-like complexes, have been identified over the last two decades [13]. The TrxG-mediated enzymatic activity involves mono-, di- and tri-methylation of the lysine 4 residue of histone H3 (H3K4me1, H3K4me2 and H3K4me3, respectively). H3K4me3 is often found enriched at transcriptionally active chromatin (euchromatin), mainly around the transcription start sites (TSSs) of gene promoters (Figure 1). Moreover, H3K4me3 can be recognized by the RNA polymerase II complex, hence, facilitating transcriptional activity at H3K4me3-marked gene promoters [24]. Importantly, the histone-methyltransferase complex MLL3/4-COMPASS-like can include additional enzymatic activities (e.g., UTX/KDM6A (Ubiquitously Tetratricopeptide Repeat X Chromosome/Lysine Demethylase 6A)) mediating the removal of histone marks like H3K27me3 [16]. Hence, binding of MLL3/4-COMPASS-like to a genomic region can additionally produce a reduced enrichment of H3K27me3, an epigenetic signature associated with decreased transcription [10,16]. SET1-COMPASS mediates the global genomic deposition of H3K4me3 in most mammalian cells, and therefore, its function is often associated with the transcriptional activation of a large number of genes (Figure 1). MLL2-COMPASS-like has been shown to be responsible for H3K4me3 deposition at promoters (Figure 1) of the homeobox genes during embryogenesis [25]. Interestingly, MLL3/4-COMPASS-like complexes can catalyze the deposition of H3K4me1 at enhancers in mammalian cells, and thus, have been recognized as an epigenetic landmark to identify putative distal regulatory enhancer sequences [26,27]. Results from several groups, however, also indicate that MLL3/4 complexes can mediate the maintenance of the H3K4me1 mark at proximal promoter regions of repressed, but poised for expression, genes (Figure 1) [28,29,30,31,32,33]. Together, these studies imply that different COMPASS and COMPASS-like complexes can be recruited to target promoter sequences in a coordinate manner to first maintain a gene silent, but poised for transcription, and subsequently, to activate its expression.

Chromatin domains with decreased enrichment of the H3K4me3 or H3K27me3 marks can also be produced and maintained in cells through the function of a selective group of enzymes with lysine demethylase activity [15,17,34]. In particular, demethylation of H3K4me3 in mammals is mediated by members of the JARID1/KDM5 (Jumonji AT Rich Interactive Domain 1/KDM5) family (JARID1/KDM5A, B and C, see Figure 1) [35], which transform H3K4me3 and H3K4me2 to H3K4me1 [36,37,38]. On the other hand, histone demethylases UTX/KDM6A and JMJD3/KDM6B (Jumonji Domain-Containing Protein D3/KDM6B) can catalyze the removal of methyl groups from H3K27me3-enriched chromatin domains (Figure 1), and therefore, counteract the silencing activity of PRC2 [39,40,41,42,43]. Together, these results support the critical role of these H3K4me3 and H3K27me3 demethylases during the control of gene activity. 

Histone methylation in chromatin also occurs at H3K9 (H3K9me1, H3K9me2 and H3K9me3), which has been found strongly associated with the formation of highly compact and transcriptionally repressed heterochromatin [44,45]. Methyltransferases (KMTs) depositing this modification (“H3K9me writers”) include SUV39H1/KMT1A (Suppressor of Variegation 3–9 Homolog 1/KMT1A), SUV39H2/KMT1B and SETDB1/ESET/KMT1E (Su(var) 3–9 and Enhancer of Zeste Domain Bifurcated 1/ERG-Associated SET Domain/KMT1E), which can mediate mono-, di- and tri-methylation (Figure 1). Alternatively, the enzymes G9A/EHMT2/KMT1C (G9A/Euchromatic Histone Methyltransferase 1/KMT1C) and GLP/EHMT1/KMT1D (G9A Related Protein/EHMT1/KMT1D), can modify this H3K9 residue but only generating H3K9me1 and H3K9me2 as final products [44,45]. The H3K9 methyl-transferase activity is counteracted by H3K9 demethylases, which can remove these repressive marks (“H3K9me1/2/3 erasers”) [46]. Among them, are LSD1/KDM1 (Lysine-Specific Demethylase 1/KDM1A); JMJD1A/KDM3A; JMJD1C/KDM3C, which can eliminate H3K9me1 or H3K9me2; JMJD2A/KDM4A; JMJD2B/KDM4B; JMJD2C/KDM4C; and JMJD2D/KDM4D, which can erase the H3K9me1, H3K9me2 and H3K9me3 marks (Figure 1). 

Chromatin organization and transcriptional activity also regulated by ATP-dependent remodelers [47,48,49]. These are multi-subunit complexes (e.g., SWI/SNF (SWItch/Sucrose Non-Fermentable)) that include a catalytic subunit (e.g., Brg1 (Brahma Related Gene 1) in the mammalian SWI/SNF) that mediates the binding and hydrolysis of ATP (ATPase activity) [50,51]. These remodelers alter chromatin structure by mobilizing nucleosomes in cis or by transferring histone octamers in an ATP-dependent manner (Figure 1). This nucleosome mobilization and/or transferring modifies the level of exposure of regulatory DNA motifs, thereby facilitating or preventing their recognition by cognate factors [52]. Whereas SWI/SNF can be specifically recruited to gene promoters by tissue-specific transcription factors [53,54,55], several reports also indicate that the targeting of SWI/SNF-related complexes can be modulated by HPTMs, including histone lysine acetylation and histone arginine methylation [56,57]. This regulation is due to the presence of bromo- and chromo-domains in subunits of SWI/SNF that can interact with these modified histone residues on the chromatin fiber [58]. 

Genomic DNA can be methylated on cytosines that are mostly followed by guanosines (CpG dinucleotides) but also by other nucleotides. The DNA methyltransferases (DNMTs) that mediate this modification belong to a well-conserved family of proteins that include both maintenance (DNMT1) and de novo (DNMT3A and DNMT3B) activities (Figure 1) [11,12]. A large body of evidence indicates that methylated CpG is associated with highly compact chromatin and reduced transcriptional activity [11,12]. DNA demethylation in mammalian cells involves the conversion from 5-methyl-CpG (5mCpG) to unmethylated CpG (Figure 1), via an intermediate transformation to 5-hydroxymethyl-CpG (5hmCpG) by the activity of the Ten Eleven Transformation (TET) family of dioxygenases [12,59,60]. Importantly, TET proteins (TET1, 2 and 3, Figure 1) form regulatory complexes with ATP-dependent chromatin remodelers like SWI/SNF as well as with histone methyltransferases and histone demethylases [61,62,63]. In recent years, accumulating evidence shows that non-CpG C-methylation can occur (at significantly lower rates than 5mCpG) along the genome of mammalian cells [64,65]. 

Several reports indicate that gene regulation in mammalian cells often requires synergistic cooperation between at least two independent epigenetic mechanisms [66,67]. This mechanistic partnership is based on the ability of proteins that “write” and “read” different epigenetic marks to form complexes at target sequences [45,46]. This further indicates that in the eukaryotic nucleus, different epigenetic mechanisms leading to chromatin remodeling and transcriptional control are functioning in a coordinated and complementary manner, hence, supporting an effective modulation of gene expression in response to physiological cues. 

## 3. Signaling Pathways Leading to the Induction of Activity-Dependent Gene-Regulation Programs during Learning and Memory Processes

**Figure 2:**
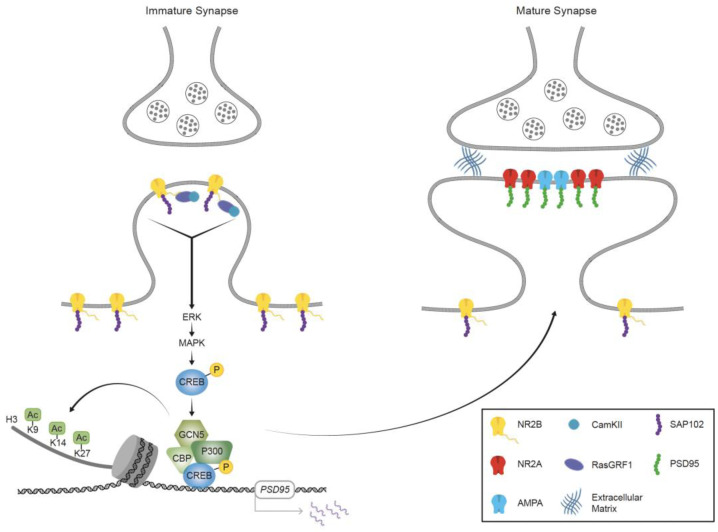
Proposed model of how the NMDAR-ERK-CREB-signaling pathway leads to activation of the PRG *PSD-95* through epigenetic mechanisms. In immature neurons, NR2B-NMDARs (important for neuronal plasticity) are enriched at the synapse via the interaction with the scaffold protein SAP102 (synapse-associated protein 102). Calcium influx through synaptic NR2B-NMDARs immediately activates CaMKII and RasGRF1 (coupled directly to the C-terminal tail of the NR2B subunit), which in turn induce the activation of ERK, MAPK and CREB (CREB-P). Activated CREB-P act as a transcription factor and recruits CBP, P300 and GCN5 to the *PSD-95* gene promoter. Subsequently, these HATs promote the acetylation of K residues in the histone H3 tail (H3K9/14/27ac) to induce gene transcription (depicted under the *PSD-95* gene). The produced PSD-95 proteins will be centrally inserted into the synapses and hold NR2A-NMDARs, displacing the previously synaptic SAP102-bound NR2B-rich receptors laterally to the extra-synaptic membrane. The synaptic NR2A-NMDARs are critical for neuronal refinement as well as for learning and memory.

In animals, memory formation and recall are essential for survival and for their adaptations to a complex and often dynamically changing environment. In humans, memory is also crucial for mental health and to define who we are, as eloquently stated by Dr. Eric Kandel: “Memory is the glue that holds our mental life together”. During memory formation, experiences prompt the activation of a selected and sparse population of neurons (engram cells) that undergo persistent physical and/or chemical changes allowing long-lasting memory. Over the past decades, important progress has been made in elucidating signaling mechanisms by which neuronal transmission leads to the induction of activity-dependent gene-regulation programs during learning [68]. A well-studied signaling mechanism during memory acquisition involves calcium influx through the predominantly synaptic-localized NMDA (N-methyl-D-aspartate) receptors (NMDARs). They trigger the activation of calcium-dependent signaling proteins that, hence, activate the extracellular signal-regulated kinase (ERK)-dependent pathway, leading to phosphorylation and nuclear localization of the transcription regulator CREB [69,70]. A particularly effective activation of the ERK-CREB signaling axis is initiated at immature synapses by the opening of the NR2B (also referred to as GluN2B or GluRε2)-containing ionotropic NMDAR channels. This allows a sustained calcium influx and an immediate activation of the calcium-sensitive signaling proteins CaMKII (Ca^2+^/calmodulin-dependent kinase II) and Ras-GRF1 (Ras protein-specific guanine nucleotide releasing factor 1) that directly interact with the C-terminal tail of the NR2B subunit (see Figure 1) [71,72,73,74,75,76,77]. Activated CREB act as a transcription factor and recruits the coactivator CBP, or its highly homologous transcriptional co-activator P300, to target genes. CBP/P300-mediated acetylation of histone H3K27ac then facilitates the recruitment of the RNA polymerase II-containing complex to mediate the transcription of hundreds of target genes [9,78,79,80]. Transcriptomic analyses indicate that the formation and consolidation of memory, including fear memory (see below), does not rely on a single event but on a dynamic process requiring several waves of transcription activation of both immediate early genes (IEGs: i.e., *Fos, Arc*) and late-response genes, including plasticity-related genes (PRGs) [81,82,83,84]. Critical PRG encoded proteins include specific subunits of NMDARs (i.e., NR2A, also referred to as GluN2A or GluRε1) and AMPARs (α-amino-3-hydroxy-5-methyl-4-isoxazole propionic acid, i.e., GluR1, also referred to as GluA1 or GluR-A), as well as the scaffold protein PSD-95 (postsynaptic density protein 95, encoded by *Dlg4* (discs large homolog 4)) that holds these glutamate receptors (as well as many additional proteins) at the postsynaptic membrane of mature synapses. This complex postsynaptic organization enables a precise temporal synaptic transmission required for memory formation and consolidation (see Figure 2) [72,85].

Despite the advances in engram research, the precise spatial-temporal location of memory and the molecular mechanisms that govern the transcriptional waves in engram cells remain poorly understood. Two recent elegant studies using genome-wide mapping strategies have begun to elucidate how changes in the epigenome [86] and in the three-dimension (3D) chromatin architecture [84] regulate transcription changes of IEGs and PRGs in engram cells. We will briefly explain several methods and assays used in these studies. Wild-type or TRAP (targeted recombination in active populations) mice (see below) were subjected to a contextual fear memory paradigm. An advantage of this contextual fear conditioning (CFC) is that a single trial enables a study in the hippocampus temporally distinct phases of memory formation, consolidation and retrieval. These phases include a basal state (also called naive; 0 h post-CFC and before acquisition), an early memory state (denoting short-term memory and early memory formation; typically, 1–2 h post-CFC), an intermediate memory state (denoting short-live long-term memory; typically, 1 d post-CFC), a late memory state (denoting long-term memory; typically, 3–7 d post-CFC) and a reactivated memory state (denoting recall; 3–7 d post-CFC + reactivating fear memory) [81,84]. In all these studies, an ideal control is used to subject animals only to the context (CTX), without applying a foot shock. Next, hippocampal or cortical tissue (i.e., to address long-term memory storage; see [87]) are obtained across different phases of CFC and subsequently analyzed to identify relevant changes within the transcriptomic profile by high-throughput RNA sequencing (RNA-seq), in the epigenomic landscape (e.g., by Chromatin immunoprecipitation coupled with high-throughput sequencing (ChIP-seq and Methylated DNA immunoprecipitation and sequencing (MeDIP-seq)), in chromatin accessibility (e.g., by the Assay for Transposase-Accessible Chromatin and sequencing (ATAC-seq)) and in the chromatin 3D organization using Chromatin-Conformation-Capture-based approaches (e.g., Hi-C). ChIP-seq data can also be used to identify putative active promoters (enriched in H3K4me3, H3K9ac, H3K27ac, H3K79me3), primed enhancers (enriched in H3K4me1), active enhancers (enriched in H3K27ac and H3K4me1) together with transcriptionally repressed genomic regions (enriched in H3K9me3, H3K27me3 and 5mCpG). Bonn and colleagues [86] begun to unravel a role for epigenetic mechanisms mediated by HPTMs and DNA methylation during short-term memory formation in hippocampal tissue (1 h-CFC) and long-term memory maintenance in cortical tissue (4 weeks-CFC). To separate NeuN^+^ (neuronal nuclear protein) neuron populations from NeuN^−^ cells (mostly glial cells) they used fluorescence-activated cell sorting (FACS). It was found that most changes in HPTMs occur during short-term memory formation and that, with the exception of H3K79me3, these differences in HPTMs enrichment correlated little with changes in gene expression. By contrast, a strong spatial-temporal correlation between associative memory with a differential DNA methylation profile was detected, including changes that were almost exclusively restricted to hippocampal neurons at 1 h-CFC and to cortical neurons at 4 weeks-CFC. Epigenetic changes also occurred in NeuN^-^ cells, suggesting a functional role for glial cells in epigenetic-mediated learning. These findings indicate that DNA methylation serves as a prominent mnemonic substrate for long–term fear memory maintenance in cortical cells. The alterations in HTPMs detected during short-term memory formation suggest that these epigenome changes play a role in neural cell population priming, sensitizing neurons and non-neuronal cells for future activity. 

In a recent study, Tsai and collaborators [84] used TRAP mice in which activated neurons expressing the IEG *Arc* are permanently fluorescence-labeled (eYFP or dVenus) in an inducible and controlled manner (e.g., [88]). This enabled the research team to specifically isolate the sparse hippocampal engram neurons. Following CFC experiments, the FACS-sorted hippocampal engram neurons were subjected to genome-wide mapping strategies (RNA-seq, ATAC-seq and promoter capture HiC (pc-HiC)) to address how the epigenome and the 3D genome architecture regulate gene transcription during early memory formation (1.5 h post-CFC), consolidation (5 d post-CFC) and recall (reactivation at 5 d-CFC). The process of identifying putative promoter and enhancer regions included ChIP-seq data from previous studies [86,89]. The data revealed that during different phases of the engram ensemble, dynamic changes in gene expression, 3D genome architecture and chromatin accessibility occur. Notable is the finding that the delayed transcriptional wave during late memory consolidation and recall correlates with a spatial reorganization of large chromatin segments that support increases in the enhancer-promoter interaction (EPI) frequency. Based on the pc-HiC data, it was proposed that engram neurons can use a subset of de novo EPIs to upregulate gene expression during recall. This finding was specifically confirmed by 3C-qPCR analyses for the genes *Grik3* (glutamate kainate 3 receptor) and *Eif3d* (eukaryotic translation initiation factor 3 subunit D), further showing that reactivated engram neurons display an increase in the interaction frequency between the promoters of these genes and their de novo long-distance enhancer. It was also found that memory acquisition leads to marked increases in chromatin accessibility at enhancers. However, this increased genome accessibility was not accompanied by significant transcriptional changes. One potential explanation for this rather surprising result is that the protein-coding transcript population analyzed in these studies [84] was very limited if compared to that of previous transcriptomic analyses in hippocampal engram neurons [83]. Thus, Marco et al. [84] performed their analyses in mostly nuclear RNA (nRNA) samples (as eYFP^+^NeuN^+^ nuclei were selected), hence, missing cytoplasmic mRNAs. Taken together, these studies indicate that it has become possible to identify specific engram cells using a combination of genome-wide mapping strategies in mouse models subjected to fear conditioning. These results show that it is critical to unravelling how the changes in the epigenome and the 3D chromatin architecture can underly physiological processes associated with learning and memory.

**Figure 3:**
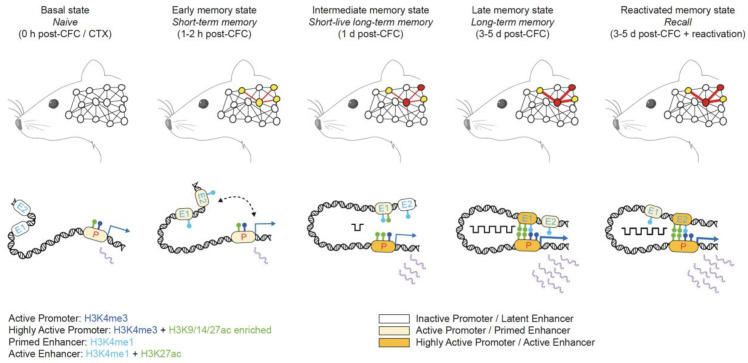
Proposed model of how temporal and spatial changes in the epigenome and the 3D chromatin architecture regulate the transcription of PRGs critical for neuroplasticity and memory. The epigenetic status of PRG promoters and enhancers along with the formation EPIs by chromatin looping are depicted across sequential stages of fear memory ensemble in hippocampal engram neurons. In the naive animal condition (i.e., basal state), basal gene expression of PRGs such as *PSD-95* is mediated by a limited activation of the promoter (P) (enrichment in H3K4me3 and limited enrichment of H3K27ac, H3K9ac and H3K14ac). During short-term memory (i.e., early memory state), enhancers (E) are primed (H3K4me1), to subsequently interact through DNA looping with their target promoters. During short-lived long-term memory (i.e., intermediate memory state)—as a result of the activation of the NMDAR-ERK-CREB-signaling pathway that mediates P300/CBP recruitment (see Figure 2)—H3K27ac becomes significantly enriched, leading to both enhancer activation (H3K4me1+H3K27ac) and increased promoter activity. Hence, there is an initial establishment of low-frequency EPIs, paralleled by an increased transcription of the PRG (depicted under the *PSD-95* gene). High-promoter and enhancer activation (reflected by high enrichment of the H3K27ac mark) during long-term memory (i.e., late-memory state) accompanies robust expression of the PRG. During recall, the delayed transcription surge of PRGs is principally mediated by a de novo EPI (depicted as enhancer 2, E2).

The above-discussed data, together with studies describing mechanisms associated with specific critical genes (e.g., PRG *PSD-95*) in hippocampal function [90,91], support a general model for engram formation, consolidation and recall (see a schematic representation in Figure 3). In this proposed model, there is a sequential and coordinated gene promoter activation that occurs along with processes of enhancer priming and subsequent enhancer activation. This activation of enhancers and promoters then mediates functional EPIs in hippocampal engram neurons during early and intermediate memory phases that may subsequently support a delayed transcriptional wave of PRGs during the late stages of fear memory consolidation and recall.

## 4. Alterations in the Epigenome and 3D Chromatin Architecture Disrupt Signaling Pathways in Neurons and Microglia Cells in AD

AD is the most prevalent neurodegenerative disease in the elderly population with a global burden of approximately 50 million people and no disease-modifying treatments available [92]. AD is characterized by pathological hallmarks, including amyloid-β plaques and tau-neurofibrillary tangles, synaptic and neuronal loss, and progressive cognitive decline. Numerous findings support the amyloid cascade hypothesis for AD, which postulates that an imbalance between production and clearance of Aβ42 and related Aβ peptides underlies the pathological and behavioral changes observed in AD patients [93]. Mechanistically, pathologic Aβ has been shown to induce synaptic failure and excitotoxicity in neurons by interacting with cell surface receptors (including NMDARs and AMPARs), scaffold proteins and intracellular signaling molecules, in addition to activating microglia and astrocytes [94]. Initial insights into the pathogenesis of AD came from genetic studies of familial autosomal dominant AD cases that identified full penetrant single-pathogenic mutations in genes regulating the production of the Aβ peptide, including the amyloid precursor protein gene (*APP*) as well as in those genes encoding the γ-secretase subunits, presenilin 1 and 2 (*PSEN1, PSEN2*). However, only a minority of the AD cases carry a pathogenic mutation in these early-onset AD genes, with the vast majority of clinical AD cases (~95%) being of late-onset and mostly sporadic or showing modest familial clustering. Among them are the *APOE* polymorphic alleles, considered, as of today, the major genetic risk factor for developing sporadic AD (i.e., carrying e4). Hence, there is a necessity to explore alternative molecular mechanisms leading to the onset and progression of AD. One approach has been to assess whether there are epigenetic alterations in the coding regions and flanking promoters of genes directly implicated in AD pathogenesis (i.e., *APP*) and in plasticity and cognition (i.e., *GluR1-2*). We then take an overview of recent evidence that describes how epigenome alterations occurring at distantly located enhancers can disrupt their ability to functionally interact with target gene promoters. As it becomes evident from several reports (e.g., [4,95]), it is also necessary to consider, in this analysis, the contribution of different subpopulations of neural cells. This is because specific cell populations obtained from AD patients and from experimental models indicate that epigenetic alterations in regulatory regions and dysfunctional EPIs are not restricted to the neuron populations (i.e., affecting synaptic plasticity genes) but are also found in other cell types in the CNS, particularly in microglia cells (i.e., affecting immune-related genes).

### 4.1. Epigenetic Alterations in Gene Loci Associated with AD Pathogenesis

To understand genetic and non-genetic associations with the onset and progression of AD, a number of researchers have analyzed, over the last two decades, the genomes of twins that are discordant for AD. One large study (11,884 twins, including 392 twin pairs in which 1 or both members had AD) indicated that ~42% of the AD patients lacked heritability [96]. In addition, immunostaining assays against the DNA methylation marker 5-methylcytosine in postmortem brain tissues from a rare pair of monozygotic twins discordant for AD demonstrated that cortical neurons, astrocytes and microglia displayed strongly reduced DNA methylation in the AD twin relative to the neurologically normal, non-demented twin [97]. The fact that the AD twin had extensive contact with pesticides in his work, strongly suggested that these epigenetic changes may have occurred in response to environmental effects. Recent reports also show that air pollution-exposed healthy urbanites (20–40 years old) and mice display reduced enrichment of repressive epigenetic marks (H3K9me2/3) along with hyperphosphorylated tau and amyloid-β plaques [98]. Hence, evidence is emerging that negative environmental factors—including environmental pollutants, infectious agents, diet and psychosocial elements—can impair brain chromatin and increase the risk of developing AD in young individuals [99,100,101].

**Figure 4:**
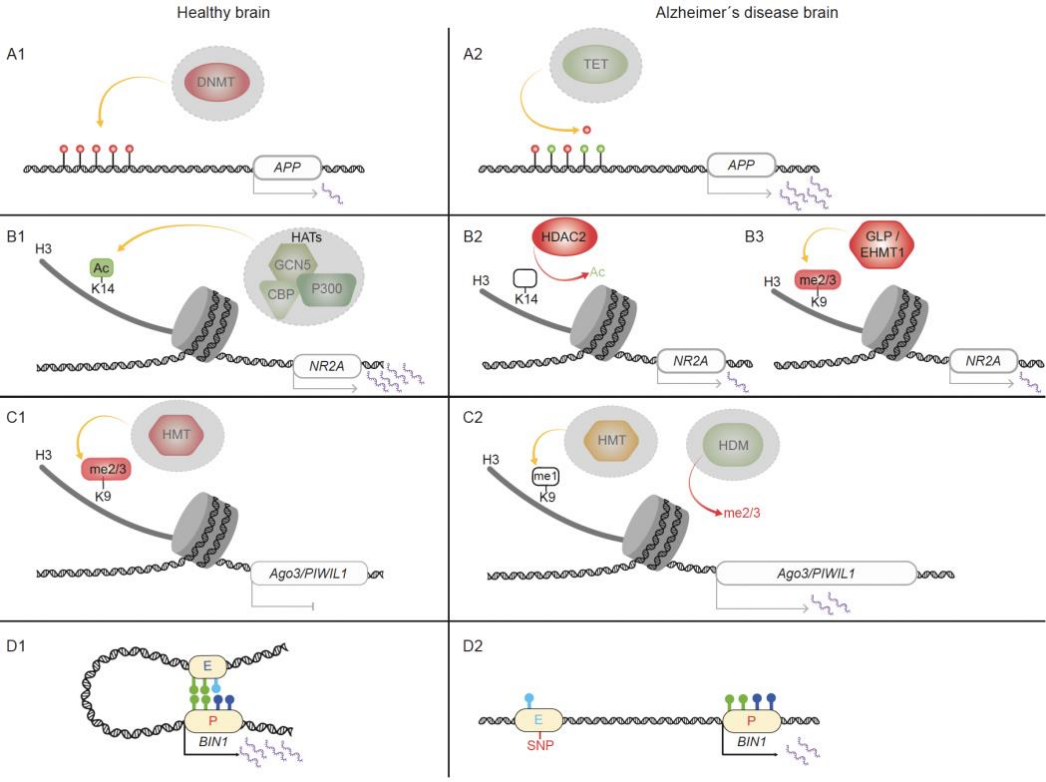
Local alterations in the epigenome and 3D chromatin architecture contribute to transcriptional dysregulation in AD. Schematic illustrations of examples of epigenetic (**A**–**C**) and chromatin remodeling (**D**) changes discovered in the brain of AD animal models and postmortem brain tissue of AD patients, compared to healthy controls. The chromatin-modifying enzymes are also depicted; those that have been demonstrated to contribute to the implicated epigenetic mechanisms are shown in bright format, while those potentially involved, are shown in shadow format. (**A**) CpG methylation (red circles) of the *APP* gene promoter in brain samples from (**A1**) healthy and (**A2**) sporadic cases of AD is depicted; unmethylated CpG (green circles) in AD is paralleled with increased *APP* mRNA levels. (**B**) H3K14ac associated with the promoter of the plasticity gene *NR2A* in brain samples from (**B1**) healthy and (**B2**,**B3**) AD mice is shown; the loss of this active mark (**B2**) and/or the enrichment in the repressive mark H3K9me2/3 (**B3**) correlates with reduced *NR2A* mRNA levels in AD. (**C**) H3K9me2/3 associated with the promoter of the normally heterochromatic and silenced gene *Ago/PIWI1* in brain samples from (**C1**) healthy and (**C2**) AD mice is depicted; the loss of this repressive mark correlates with the induction of *Ago/PIWI1* mRNA levels in AD mice and AD patients. (**D**) The *BIN1* gene promoter and its long-distance enhancer element in microglia derived from brain samples from (**D1**) healthy and (**D2**) AD mice, are shown; loss of this EPI in AD correlates with reduced expression of *BIN1* mRNA. The location of the AD risk variant rs6733839 is also depicted in the enhancer sequence. For more details, see text.

These and other studies have led to the question of whether epigenetic alterations at promoters of AD-associated genes can lead to late-onset AD. Over a decade of epigenome-wide studies focusing on the DNA methylation status at causal and risk genes for AD have provided insights into this important question. Particularly interesting are results related to the methylation status of the *APP* gene promoter that was found to include several methylated CpGs in control subjects that are lost in AD patients. Thus, DNA methylation studies at specific gene locus in postmortem brains (mixed-cell populations) [102,103] or analyzed by epigenome-wide association studies (EWAS) on sorted neuronal and non-neuronal (mostly glia) nuclei [95], revealed that reduced CpG methylation on the *APP* gene promoter in both neurons and glial cells in sporadic cases of AD accompanies increased *APP* gene expression (see Figure 4A). This result is in agreement with other studies showing that duplication of the wild-type *APP* gene causes AD due to an overexpression of APP that in turn results in the generation of excessive Aβ [104,105,106].

It has been also important to determine that there is a correlation between the DNA methylation profile and the Braak stages (after correcting for age and gender), where the *APP* gene promoter is progressively hypomethylated during later stages of AD [95]. These data suggest that progressive loss of DNA methylation at the *APP* gene promoter, and hence, an epigenetically mediated activation of the *APP* gene, plays a critical role in driving late-onset AD. Previous methylome profiling studies, however, on purified neural cell populations [95] as well as in hundreds of bulk brain tissue samples [107,108,109] of AD patients, did not bring conclusive evidence to support epigenetic dysregulation in the gene body and/or the promoter regions of other key AD genes involved in the formation of neurofibrillary tangles (*GSK3B, MAPT*) and regulating the production of Aβ peptide (*BACE1, PSEN1, PSEN2*). Intriguingly, these studies concluded that changes in DNA methylation are occurring at genes associated with other important processes including inflammation, neurotransmitter homeostasis and transport (e.g., *MCF2L, ANK1, HOX3A, MAP2, LRRC8B, STK32C, S100B, KIF26A*) [95,109]. Some of these changes in DNA methylation have been found to be restricted to either neurons (i.e., HOX3A) or glia (i.e., *ANK1*), underscoring the complex interplay between a neuronal versus glial epigenetic burden in the AD brain. Future careful examination of the relationship between DNA methylation and gene expression level in AD-related samples will continue to be necessary as several controversial results remain unclarified in the field (e.g., [110,111]). 

Studies focusing on HPTMs also support the role of an aberrant epigenetic regulation in AD. Thus, a recent comprehensive multi-omics analysis—integrating transcriptomic, proteomic and epigenomic analyses of postmortem human brains from AD patients (and comparing to brains of old and young control subjects)—revealed global gains in the active marks H3K27ac and H3K9ac [112]. Moreover, by correlating ChIP-seq and RNA-seq analyses the authors further showed that these marks are associated with transcription-, chromatin- and disease-related pathways [112]. 

Using the CK-p25 mouse model (overexpressing p25 (a truncated version of p35) that aberrantly activates cyclin-dependent kinase 5 (Cdk5)), compelling studies by the Tsai laboratory have provided mechanistic insights about the epigenetic dysregulation of histone acetylation that contributes to impaired synaptic plasticity, neurodegeneration and cognitive decline in AD [113,114,115,116,117,118]. In both AD patients and AD mouse models, aberrant synaptic plasticity is associated with a reduction in the expression of genes (mRNA and protein) implicated in learning and memory and synaptic plasticity. This reduced transcription is accompanied by several local epigenetic changes at the promoters of these genes, for example, due to diminished epigenetic activation by CBP/P300 HATs or due to an epigenetic gene suppression mediated by promoter-bound HDACs. Thus, immunostaining assays revealed increased global nuclear levels of HDAC2 in neurons of brain samples from postmortem human sporadic AD patients and several AD mouse models, including CK-p25, 5xFAD (expressing human *APP* and *PSEN1* transgenes with a total of five AD-linked mutations) [115] and AβPPswe/PS-1 (also termed APP/PSEN1, expressing a chimeric mutant mouse/human APP and a mutant human PS1) [119]. As HDAC2 has been shown to interact with the promoter region of many genes involved in memory and synaptic plasticity (*Arc, Bdnf, GluR1, NR2A, CaMKII, PSD-95*) [114], it was also determined if HDAC2 enrichment is significantly higher at these gene promoters in CK-p25 mice brain [115]. ChIP assays showed increased binding of HDAC2 to genes with critical roles in learning and memory (i.e., *Arc*, *Bdnf*) and synaptic plasticity (i.e., *GluR1*, *GluR2*, *NR2A* and *NR2B*). These studies also confirmed that decreased levels of active histone acetylation marks (e.g., H3K14ac, H4K12ac) accompanied this reduced gene expression profile in CK-p25 mice (see Figure 4B1,2). These results suggest that in AD, HDAC2 (and likely other HDAC family members) is capable of erasing histone acetylation at these actively transcribed genes. Since histone acetylation-mediated epigenetic control is highly dynamic, it was also determined that a knock-down of HDAC2 [115] or treatment with diverse HDAC inhibitors targeting HDAC2 [113,116,117] rescues pathologic cognitive deficits in AD mice, promoting neuroplasticity-related gene expression, reinstating morphological alterations and synaptic plasticity and restoring memory deficits. Together, these results indicate that transcriptional repression of neuroplasticity genes associated with decreased histone H3 and H4 acetylation may significantly contribute to AD pathology and cognitive impairment. 

These results have strongly advocated for the development of effective therapeutic strategies using selective HDAC2 (or to other HDACs) inhibitors that restore an active promoter epigenetic state (H3/H4 acetylation) of critical neuroplasticity genes. Thus, several studies have shown the beneficial effects of a wide variety of HDAC (i.e., HDAC1, HDAC4 and HDAC6) inhibitors on learning and memory, by not only reactivating plasticity but also by modulating Tau function and oxidative DNA repair in AD [116,117,120,121,122,123]. Moreover, during the last decade, small chromatin-modifying molecules, known as epidrugs, have been generated. Recent studies include epigenetic screens of selective small molecule libraries seeking to identify molecules that modulate the activity of histone-modifying enzymes, or alternatively, that can target critical domains (e.g., bromodomains and chromodomains) present in epigenetic readers [124]. These screens have been carried out in experimental models of frontotemporal dementia (FTD) (i.e., expressing hexanucleotide repeat expansion in the *C9ORF72* gene), the second most common dementia after AD [125] that exhibit alterations in the repressive marks H3K9me3, H3K9me27 and DNA methylation in neurons and astrocytes [126,127,128,129]. In some of these studies, treatment with a specific class of epidrugs (i.e., JQ1 and PFI-1, both members of the bromodomain and extra-terminal domain (BET) inhibitor family) was shown to restore gene transcription in mouse and human iPSC-derived neurons, and moreover, to ameliorate cognitive deficits in FTD mice [130,131]. However, in a comparable JQ1 treatment it was shown that this BET inhibitor can inhibit non-spatial learning in wild-type mice [132], indicating that additional studies that precisely determine the genomic regions affected by JQ1 treatment are required. 

**Scheme 1:**
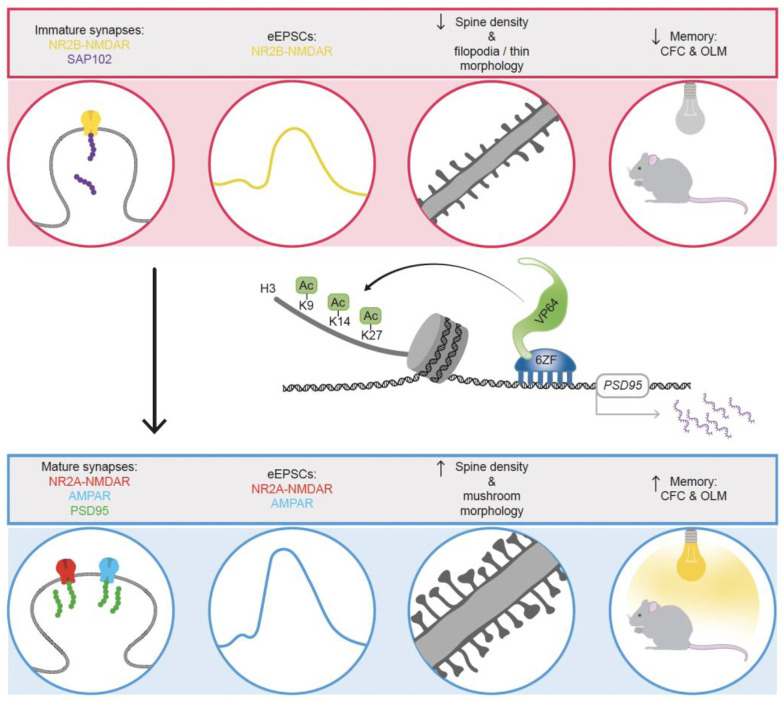
Epigenetic editing at target genes as a novel therapeutic strategy. In AD, the expression of PSD-95 is significantly reduced [137–139]. To restore endogenous *PSD-95* gene expression in AD, an epigenetic editing tool was designed where a specific zinc finger DNA-binding domain targeting the *Dlg4*/*PSD95* gene promoter was fused to the epigenetic activating effector domain VP64 [91]. Transduction of this epi-editor induced local epigenetic reprogramming, increased PSD-95 mRNA and protein levels, and impacted various plasticity-associated processes, including synaptic transmission and dendritic spine density. Notably, viral-mediated delivery of the epi-editor to hippocampal neurons prevented (pre-symptomatic treatment) and even restored (symptomatic treatment) memory deficits in the AD mice model AβPPswe/PS-1. Given that the target sequence identified at the *PSD-95* gene promoter is 100% identical among mice, rats and humans, these findings establish epigenetic editing as a potential therapy to treat AD and other human neurological disorders displaying *PSD-95* haploinsufficiency and/or dysfunction, including Huntington´s disease (HD), amyotrophic lateral sclerosis (ALS), intellectual disability, schizophrenia and autism spectrum disorders [85,137,138,139,140,141,142,143].

Another successful and potentially therapeutical approach has been to selectively modify chromatin by using epigenome editing tools, that hence, modulate the expression of a specific target gene in a precise manner [133,134,135]. The scaffolding protein PSD-95, encoded by the gene *DLG4*, was shown to be an ideal target for this type of approach as it is a critical protein for synaptic plasticity, dendritic spine stabilization and learning and memory (see above). Additionally, PSD-95 expression has been found reduced in sporadic AD patients [136] and in AD mouse models [91,137]. Epigenetic editing of the *Dlg4/PSD95* gene promoter led to a local epigenetic reprogramming and increased PSD-95 expression, impacting various plasticity-associated processes, and importantly, restoring memory deficits in the AD mice model AβPPswe/PS-1 [91] (for more details on this topic see Scheme 1).

Epigenetic gene regulation can also be mediated by histone methylation. Thus, several studies have focused on the role of the repressive marks H3K9me2 and H3K9me3 that significantly contribute to regulating euchromatin and heterochromatin formation and maintenance in the nucleus. Enrichment of H3K9me2 and H3K9me3 can result in the repression of gene transcription in both euchromatic and facultative heterochromatic regions. These marks can also contribute to maintaining genome stability (by silencing repetitive DNA elements and transposons) and protecting DNA from damage [9,44,144,145,146]. Recent studies have documented altered H3K9me2 and H3K9me3 levels in the brains of patients and experimental models of AD. Intriguingly, in some studies, H3K9me2/3 expression in the nuclei was found to decrease [147,148], whereas in other studies these modifications were shown to be increased [149,150]. Several reasons may explain these seemingly opposite results, including differences in human and mouse brain regions and in the cell types that were examined. In addition, there appear to be significant differences in the methods used for human (postmortem) sample preparation and in the approaches followed to detect H3K9me2. Finally, it is also important to consider that in most cases only limited information (if any information at all) is available about these AD patients, including their genetic background, clinical development, and the presence of specific pathological hallmarks. Together, these uncertainties increase the difficulty of precisely comparing the different analyses carried out in the field and generating strong conclusions from the results. 

A recent study [150] reports elevated H3K9me2 levels in the prefrontal cortex of postmortem human AD patient samples (H3K9me2 detected by western blot) and of 5xFAD mice (H3K9me2 detected by western blot and immunostaining). Moreover, ChIP assays on 5xFAD brain samples further confirmed that an increased enrichment of H3K9me2 occurs at the promoters of genes coding for AMPAR (GluR2/GluA2) and NMDAR (NR2B/GluN2B) subunits, concomitantly with decreased expression (detected at mRNA and protein levels) and function of these receptors (measured by electrophysiology) (see Figure 4B3). Additionally, it was found that the H3K9 di-methyltransferase EHMT1 (or G9a-like protein, GLP) is upregulated in the prefrontal cortex of the 5xFAD mice. Notably, treatment with the EHMT1/2 (G9a/GLP) inhibitor BIX01294 restored transcription, protein expression and function of AMPARs and NMDARs, and importantly, rescued memory deficits in this AD mouse model [150]. These findings indicate that an EHMT1-mediated increase in histone H3K9 methylation can significantly contribute to transcriptional repression of critical neuroplasticity genes, and hence, to the pathology and cognitive decline in AD. The study also suggests that treatment with specific epidrugs may function as an effective therapeutic strategy to ameliorate, and potentially reverse, memory decline in AD. This conclusion is supported by a parallel study where treatment with the EHMT1/2 inhibitor UNC0642 was capable of restoring cognition parameters in animal models, together with reducing the expression of inflammatory markers and increasing the levels of neurotrophic factors [151].

In another study, Feany and collaborators [147], analyzing FACS-purified neurons of hippocampal tissue obtained from AD patients, also detected a strong reduction of global H3K9me2 levels. Interestingly, they found that the expression of euchromatic genes remained largely unchanged between brain samples from control and AD subjects. Moreover, it was determined that this depletion of H3K9me2 in AD affects the expression of genes that are mostly located at genomic regions silenced by heterochromatin in normal hippocampal cells. Notably, widespread transcriptional increases in non-coding genes (including *piwi* transcripts; see below) that are normally silenced in controls, were detected in AD patient brain samples. This heterochromatin loss and aberrant gene expression were found to be conserved among mouse and *Drosophila* tauopathy models [147]. Thus, ChIP-seq assays in tau transgenic flies revealed a strong loss of H3K9me2 enrichment at genes like *Ago3* (which is homologous to the human gene *PIWIL1*), concomitant with upregulated gene transcription (see Figure 4C). In a posterior study, Frost and colleagues [152] demonstrated that decondensation of constitutive heterochromatin occurs concomitant with transcriptional activation of transposable elements in the brains of postmortem human AD patient samples as well as in brain cells of fly models. Mechanistically, the authors proposed an interesting model where tau-induced heterochromatin de-condensation facilitates active transcription of transposable elements and that tau-induced depletion of *piwi* and *piwi*-interacting RNAs (piRNAs) enables the transcripts from the transposable elements to remain elevated. How a pathologic tau precisely causes a global loss of heterochromatin-dependent silencing is still not understood. A strong role of DNA damage, induced by excessive oxidative stress, has been postulated [147,153]. Similarly, it has been proposed that the loss of the physiological role of endogenous tau, which directly binds and regulates H3K9me3-rich pericentromeric heterochromatin integrity in neurons, is an important component [148,154].

### 4.2. Epigenetic Changes Impact Promoter–Enhancer Interactions in AD

As discussed above, the vast majority of AD cases cannot be explained by pathogenic mutations occurring in AD protein-coding genes. In the last 10 years, next-generation sequencing (NGS) and genome-wide association studies (GWAS) have led to the identification of numerous low penetrance predisposing genetic mutations and single nucleotide polymorphisms (SNPs) at more than 40 susceptibility loci associated with late-onset AD [92,155,156]. Identification of AD-associated variants in a specific-coding gene has only been established for a few loci (e.g., *APP, TREM2, TREML2, PLCG2, UNC5C, ADAM10, AKAP9*). Intriguingly, cell type-specific gene expression profiling and analysis of related biological processes show that several of these genetic variants are selectively expressed in microglia and involved in immune responses and lipid metabolism (e.g., *TREM2, TREML2, PLCG2*) [155,157]. 

In the past few years, comprehensive genome- and epigenome-wide maps have been created to find that many AD susceptibility loci lie in putative cell-type-specific enhancer elements. Initial support to this novel concept came from a study comparing transcriptional and chromatin states between hippocampal samples from the AD-like mouse model CK-p25 and humans, followed by a close examination of the enrichment of AD-related SNPs within conserved enhancers [118]. Briefly, transcription and chromatin dynamics (measured by ChIP-seq to identify putative primed/active promoters and enhancers; see above) were examined across early (2 weeks) and late (6 weeks after p25 induction) stages of pathology in CK-p25 mice. In agreement with previous studies, it was determined that during the late stages of AD pathology genes involved in synaptic plasticity and learning were down-regulated, concurrent with reductions in the activity of their assigned promoter and enhancer regions [115]. In contrast, during the early stages of AD pathology, immune-response genes were found upregulated, concomitant with increases in the activity of their cognate-regulatory genomic regions. Researchers then mapped orthologous coding and non-coding regions between mouse and human hippocampal samples, identifying significant human-to-mouse conservation of epigenomic signatures and gene expression profiles. Notable was the result indicating that AD-associated genetic variants (i.e., *PICALM, BIN1, NPP5D, CELF1/SPI1, PTK2B*) were specifically enriched at enhancer orthologues that displayed increased activity, implicating a role of immune-related processes in AD predisposition [118]. These results in the CK-p25 mice also indicated that epigenetic changes (without SNPs) in regulatory regions controlling immune processes and synaptic plasticity can contribute to AD pathology. 

In two recent studies, using postmortem non-diseased human brains, chromatin interactions between enhancers and promoters were established by subjecting specific cell-type populations to pc-HiC analysis [5], in combination with ATAC-seq and PLAC-seq (proximity ligation-assisted ChIP that captures EPIs with active H3K4me3 bearing promoters) [4]. Epigenomic annotations were used to identify putative primed/active promoters and enhancers (as described above). As expected, chromatin loops were detected between active promoters and distal regulatory regions in neurons, microglia, astrocytes and oligodendrocytes [4,5]. By examining the genomic location of disease-associated GWAS variants, it was determined that AD variants were only enriched in microglia enhancers (i.e., *BIN1, PICALM, SORL1, SPI1*). This is a particularly intriguing result as most polymorphisms associated with psychiatric and neurological disorders (e.g., autism, schizophrenia, neuroticism) have been located in neuronal enhancers and promoters, with few of the SNPs located in glial promoters (see also [158]). Integration of the genome-wide studies further indicated that *BIN1* is a microglia-specific enhancer as it interacts with the *BIN1* promoter and is specifically detected in microglia cells but not in neurons, astrocytes or oligodendrocytes [4]. Importantly, this *BIN1* microglia-specific enhancer also harbors the AD risk variant rs6733839, which has the second highest AD-risk score after *APOE*. To determine whether this microglia-specific enhancer is functional, a CRISPR/Cas9-mediated deletion of a 363-bp region harboring rs6733839 in human iPSC lines was performed, and these cells were then differentiated to microglia, astrocytes and neurons. Interestingly, the edition of this regulatory region leads to a microglia-specific reduction in *BIN1* mRNA and protein expression [4]. Although the study did not demonstrate the functionality of the specific SNP (e.g., editing by CRISPR), the data support the intriguing possibility that AD risk variants located in enhancer regions could change gene expression by altering the interaction between enhancer-promoter (Figure 4D).

## 5. Concluding Remarks 

During the last decades, several signaling pathways have been shown to induce activity-dependent gene programs in learning and memory processes. More recently, basic mechanisms operating during the spatial-temporal location of memory and governing the transcription waves in engram cells have been defined. Critical in these advances has been the integration of transcriptomic and epigenomic analyses together with 3D chromatin mapping in mouse models. Applying comparable multi-omics analyses in both animal models and human postmortem brain tissue of patients, researchers in the field have begun to reveal how global and local changes in the epigenome and 3D chromatin architecture contribute to transcriptional dysregulation in AD and, very important, how this impacts the onset and progression of the disease. These studies also underscore the complex interplay between a neuronal versus glial epigenetic-dependent control of gene expression in the AD brain. This appears to be also valid for other neurodegenerative diseases, including amyotrophic lateral sclerosis (ALS), Huntington´s disease (HD) and Parkinson´s disease (PD), as it has been shown that multiple global and local changes in histone marks contribute to transcriptional dysregulation in these diseases (reviewed in [1,3]. Nevertheless, in the case of these latter diseases, systematic multi-omics analyses on animal models and patient samples remain insufficient. For a deep understanding of the molecular mechanisms underlying pathology in these diverse neurodegenerative diseases, it would be then critical to determine whether common epigenetic and chromatin-associated mechanisms are dysregulated. This may provide an opportunity to develop more universal therapeutic approaches to mitigate disease progression and symptoms that appear shared by many of these diseases. Since multiple types of brain cells participate during the onset and progression of the pathologic signs identified in these diseases, significant advances may be reached by applying novel techniques like immunoGAM [159,160]. This technique is an extension of the previous Genome Architecture Mapping (GAM) and allows a wide 3D mapping of the chromatin topology in specific brain cell types and brain tissue sections from animal models. Likely in the future, this type of approach will permit these analyses in postmortem tissue samples from patients. 

Our increased understanding of the role of specific genes and regulatory pathways in brain cells has led to the development of potential new therapeutic strategies. We envision that in the future, epigenetic editing tools will be used to restore the expression of specific genes that are fundamental for the maintenance of neuronal connectivity, plasticity and memory. Moreover, it is reasonable to speculate that targeting fundamental plasticity genes, such as *PSD-95,* might not only be beneficial for treating AD, but also other brain disorders including HD and ALS, where the expression of this critical postsynaptic protein is also reduced (see Scheme 1). While epigenetic editing constructs are good candidates for developing new gene therapy strategies, the need for adequate viral delivery approaches has hampered the progress in bringing (epi)genome therapies to clinical trials. However, the recent massive use of adenoviruses for the delivery of SARS/CoV-2 genes to patients as a vaccination strategy throughout the planet may rapidly change this aspect, given the reduced negative collateral signs observed so far. Alternative, but complementary, studies strongly advocate that specific epidrugs may function as an effective therapeutic strategy to ameliorate, and potentially reverse, memory decline in AD and FTD. How these epidrugs affect the epigenome landscape in diverse brain regions, however, remains a critical issue in the field and still requires extensive additional preclinical research.
